# DEN-IM: dengue virus genotyping from amplicon and shotgun metagenomic sequencing

**DOI:** 10.1099/mgen.0.000328

**Published:** 2020-03-05

**Authors:** Catarina I. Mendes, Erley Lizarazo, Miguel P. Machado, Diogo N. Silva, Adriana Tami, Mário Ramirez, Natacha Couto, John W. A. Rossen, João A. Carriço

**Affiliations:** ^1^​ Instituto de Microbiologia, Instituto de Medicina Molecular, Faculdade de Medicina, Universidade de Lisboa, Lisboa, Portugal; ^2^​ University of Groningen, University Medical Center Groningen, Department of Medical Microbiology and Infection Prevention, Groningen, The Netherlands

**Keywords:** dengue virus, surveillance, metagenomics, reproducibility, workflow, containerization, scalability

## Abstract

Dengue virus (DENV) represents a public health threat and economic burden in affected countries. The availability of genomic data is key to understanding viral evolution and dynamics, supporting improved control strategies. Currently, the use of high-throughput sequencing (HTS) technologies, which can be applied both directly to patient samples (shotgun metagenomics) and to PCR-amplified viral sequences (amplicon sequencing), is potentially the most informative approach to monitor viral dissemination and genetic diversity by providing, in a single methodological step, identification and characterization of the whole viral genome at the nucleotide level. Despite many advantages, these technologies require bioinformatics expertise and appropriate infrastructure for the analysis and interpretation of the resulting data. In addition, the many software solutions available can hamper the reproducibility and comparison of results. Here we present DEN-IM, a one-stop, user-friendly, containerized and reproducible workflow for the analysis of DENV short-read sequencing data from both amplicon and shotgun metagenomics approaches. It is able to infer the DENV coding sequence (CDS), identify the serotype and genotype, and generate a phylogenetic tree. It can easily be run on any UNIX-like system, from local machines to high-performance computing clusters, performing a comprehensive analysis without the requirement for extensive bioinformatics expertise. Using DEN-IM, we successfully analysed two types of DENV datasets. The first comprised 25 shotgun metagenomic sequencing samples from patients with variable serotypes and genotypes, including an *in vitro* spiked sample containing the four known serotypes. The second consisted of 106 paired-end and 76 single-end amplicon sequences of DENV 3 genotype III and DENV 1 genotype I, respectively, where DEN-IM allowed detection of the intra-genotype diversity. The DEN-IM workflow, parameters and execution configuration files, and documentation are freely available at https://github.com/B-UMMI/DEN-IM).

## Data Summary

1. The Supplementary Material and tables are available at Figshare under https://doi.org/10.6084/m9.figshare.11316599.v1.

2. The 106 DENV-3 amplicon sequencing paired-end short-read datasets are available under BioProject PRJNA394021 and the 78 DENV-1 amplicon sequencing single-end short-read datasets are available under BioProject PRJNA321963. The 25 shotgun metagenomics dataset is available under BioProject PRJNA474413. The accession numbers for all the samples in the shotgun metagenomics dataset are available in the Supplementary Material (https://doi.org/10.6084/m9.figshare.11316599.v1).

3. The accession numbers for the 41 samples belonging to the Zika virus, Chikungunya virus and yellow fever virus amplicon and shotgun metagenomic datasets are available in the Supplementary Material (https://doi.org/10.6084/m9.figshare.11316599.v1).

4. DEN-IM reports for the analysed datasets are available at Figshare (https://doi.org/10.6084/m9.figshare.11316599.v1).

5. Phylogeny inference trees for the dengue virus typing database are available at Figshare (https://doi.org/10.6084/m9.figshare.11316599.v1).

6. Code for the DEN-IM workflow is available at https://github.com/B-UMMI/DEN-IM and documentation, including step-by-step tutorials, is available at https://github.com/B-UMMI/DEN-IM/wiki).

**Impact Statement**

The risk of exposure to DENV is increasing, not only because of travel to endemic regions, but also due to the broader dissemination of the mosquito, making the burden of dengue very significant. The decreasing costs and wider availability of high-throughput (HTS) sequencing make it an ideal technology to monitor dengue virus’s (DENV’s) transmission. Metagenomics approaches decrease the time required to obtain nearly complete DENV sequences without the need for time-consuming viral culture through the direct processing and sequencing of patient samples. A ready-to-use bioinformatics workflow, enabling the reproducible analysis of DENV, is therefore particularly relevant for the development of a straightforward HTS workflow. DEN-IM was designed to perform a comprehensive analysis in order to generate either assemblies or consensus of full DENV coding sequences and to identify their serotype and genotype. DEN-IM can also detect all four DENV serotypes and the respective genotypes present in a spiked sample, raising the possibility that DEN-IM can play a role in the identification of co-infection cases whose prevalence is increasingly perceived in highly endemic areas. Although it is ready to use, the DEN-IM workflow can be easily customized to the user’s needs. DEN-IM enables reproducible and collaborative research, and is accessible to a wide group of researchers, regardless of their computational expertise and the resources available.

## Introduction

The dengue virus (DENV), a single-stranded positive-sense RNA virus belonging to the genus *Flavivirus*, is one of the most prevalent arboviruses and is mainly concentrated in tropical and subtropical regions. Infection with DENV results in symptoms ranging from mild fever to haemorrhagic fever and shock syndrome [1]. Transmission to humans occurs through the bite of *Aedes* mosquitoes, namely *Aedes aegypti* and *Aedes albopictus* [2]. In 2010, it was predicted that the burden of dengue disease would reach 390 million cases per year worldwide [3]. Dengue has the greatest clinical significance of any arbovirus because of the high morbidity and mortality associated with it [4]. DENV is a significant public health challenge in countries where infection is endemic due to the high health and economic burden. Despite the emergence of novel therapies and ecological strategies to control the mosquito vector, there are still important knowledge gaps concerning the virus biology and its epidemiology [2].

The viral genome of ~11 000 nucleotides consists of a coding sequence (CDS) of approximately 10.2 Kb that is translated into a single polyprotein encoding three structural proteins (capsid, C; premembrane, prM; envelope, E) and seven non-structural proteins (NS1, NS2A, NS2B, NS3, NS4A, NS4B and NS5). Additionally, the genome contains two non-coding regions (NCRs) at its 5′ and 3′ ends [5].

DENV can be classified into four serotypes (1, 2, 3 and 4), differing from each other by 25–40 % at the amino acid level. They are further classified into genotypes that vary by up to 3 % at the amino acid level [2]. The DENV-1 serotype comprises five genotypes (I–V), DENV-2 comprises six (I–VI, also named American, Cosmopolitan, Asian-American, Asian II, Asian I and Sylvatic), DENV-3 comprises four (I–III and V) and DENV-4 also comprises four (I–IV).

Although real-time reverse transcription polymerase chain reaction (RT-PCR) will probably remain the front line tool in dengue aetiological diagnosis, the implementation of a surveillance system relying on high-throughput sequencing (HTS) technologies allows the simultaneous identification and characterization by serotyping and genotyping of DENV cases at the nucleotide level in a single methodological step. Due to the high sensitivity of these technologies, previous studies have shown that viral sequences can be obtained directly from patient sera using a shotgun metagenomics approach [6]. Alternatively, HTS can be used in an amplicon sequencing approach in which a PCR step is used to preamplify viral sequences before sequencing. In recent years, HTS has been used successfully as a tool for the identification of DENV directly from clinical samples with as few as ~2 reads in a total of 10^6^ reads [6, 7]. This also allows the rapid identification of the serotype and genotype, which is important for disease management, as the genotype may be associated with disease outcome [8].

Several initiatives aim to facilitate the identification of the DENV serotype and genotype from HTS data. The Genome Detective project (https://www.genomedetective.com/) offers the online Dengue Typing Tool [9] (https://www.genomedetective.com/app/typingtool/dengue/), which relies on blast and phylogenetic methods in order to identify the closest serotype and genotype, but it requires assembled genomes in the fasta format as input. The same project also offers the Genome Detective Typing Tool (https://www.genomedetective.com/app/typingtool/virus/) [10], which identifies which viruses are present in an HTS sample and provides their assembled genome. Additionally, several tools are available for viral read identification and assembly, such as VIP [11], virusTAP [12] and drVM [13], but none performs genotyping of the identified reads.

We developed DEN-IM as a ready-to-use, one-stop, reproducible bioinformatic analysis workflow for the processing and phylogenetic analysis of DENV using short-read HTS data. DEN-IM is implemented in Nextflow [14], workflow management software that uses Docker (https://www.docker.com) containers with preinstalled software for all the workflow tools. The DEN-IM workflow, as well as parameters and documentation, are available at https://github.com/B-UMMI/DEN-IM.

### The DEN-IM workflow

**Fig. 1.**
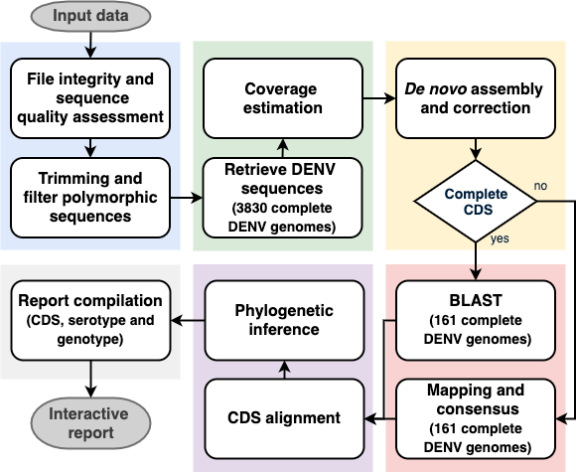
The DEN-IM workflow separated into five different components. The raw sequencing reads are provided as input to the first block (in blue), responsible for quality control and elimination of low-quality reads and sequences. After successful preprocessing of the reads, these enter the second block (green) for retrieval of the DENV reads using the mapping database of 3858 complete DENV genomes as a reference. This block also provides an initial estimate of the sequencing depth. After the *de novo* assembly and assembly correction block (yellow), the CDSs are retrieved and then classified with the reduced-complexity DENV typing database containing 161 sequences representing the known diversity of DENV serotypes and genotypes (red). If a complete CDS fails to be assembled, the reads are mapped against the DENV typing database and a consensus sequence is obtained for classification and phylogenetic inference. All CDSs are aligned and compared in a phylogenetic analysis (purple). Lastly, a report is compiled (grey) with the results of all the blocks of the workflow.

DEN-IM is a user-friendly automated workflow enabling the analysis of amplicon and shotgun metagenomics data for the identification, serotyping, genotyping and phylogenetic analysis of DENV, as represented in Fig. 1, accepting raw short-read sequencing data (fastq files) as input, single-end or paired-end, and informing the user with an interactive and comprehensive HTML report (see Fig. S1 available in the online version of this article), as well as providing output files of the whole pipeline.

It is implemented in Nextflow, a workflow management system that allows the effortless deployment and execution of complex distributed computational workflows in any UNIX-based system, from local machines to high-performance computing clusters (HPCs) with a container engine installation, such as Docker (https://www.docker.com/), Shifter [15] or Singularity [16]. DEN-IM integrates Docker-containerized images, which are compatible with other container engines, for all the tools necessary for its execution, ensuring reproducibility and the tracking of both software code and version, regardless of the operating system used.

Users can customize the workflow execution either by using command line options or by modifying the simple plain-text configuration files. To make the execution of the workflow as simple as possible, a set of default parameters and directives is provided. An exhaustive description of each parameter is available in the Supplementary Material (see DEN-IM_Supplemental_material.pdf, Workflow parameters).

The local installation of the DEN-IM workflow, including the Docker containers with all the tools needed and the curated DENV database, requires 15 gigabytes (GB) of free disk space. The minimum requirements to execute the workflow are at least 5 GB of memory and four CPUs. The disk space required for execution depends greatly on the size of the input data, but for the datasets used in this article, DEN-IM generates approximately 5 GB of data per GB input data.

DEN-IM workflow can be divided into the following components.

#### Quality control and trimming

The quality control (QC) and trimming block starts with a process to verify the integrity of the input data. If the sequencing files are corrupted, the execution of the analysis of that sample is terminated. The sequences are then processed by FastQC https://www.bioinformatics.babraham.ac.uk/projects/fastqc/), version 0.11.7) to determine the quality of the individual base pairs of the raw data files. The low-quality bases and adapter sequences are trimmed by Trimmomatic (version 0.36) [16]. In addition, reads with a read length shorter than 55 nucleotides after trimming are removed from further analyses. Lastly, the low-complexity sequences, containing over 50 % of poly-A, poly-N or poly-T nucleotides, are filtered out of the raw data using PrinSeq [17] (version 0.10.4).

#### Retrieval of DENV sequences

In the second step, DENV sequences are selected from the sample using Bowtie 2 [18] (version 2.2.9) and SAMtools (version 1.4.1) [19]. As a reference, we provide the DENV mapping database, a curated DENV database composed of 3858 complete DENV genomes. An in-depth description of this database is available in the Supplementary Material (see DEN-IM_Supplemental_material.pdf, Dengue virus reference databases). In paired-end data, a permissive approach is followed by allowing for mates to be kept in the sample even when only one read maps to the database in order to keep as many DENV-derived reads as possible. The output of this block is a set of processed reads of putative DENV origin.

#### Assembly

DEN-IM applies a two-assembler approach to generate assemblies of the DENV CDSs. To obtain a high confidence assembly, the processed reads are first *de novo* assembled with SPAdes (version 3.12.0) [20]. If the full CDS fails to be assembled into a single contig, the data are reassembled with the megahit assembler (version 1.1.3) [21], a more permissive assembler developed to retrieve longer sequences from metagenomics data. The resulting assemblies are corrected with Pilon (version 1.22) [22] after mapping the processed reads to the assemblies with Bowtie 2.

If more than one complete CDS is present in a sample, each of the sequences will follow the rest of the DEN-IM workflow independently. If no full CDS is assembled with either SPAdes or megahit, the processed reads are passed on to the next module for consensus generation by mapping, effectively constituting DEN-IM′s two-pronged approach using both assemblers and mapping.

#### Typing

For each complete DENV CDS, the serotype and genotype is determined with the Seq_Typing tool (https://github.com/B-UMMI/seq_typing, version 2.0) [23] using blast [24] and the custom DENV typing database containing 161 complete sequences (see DEN-IM_Supplemental_material.pdf, Dengue virus reference databases). The tool determines which reference sequence is most closely related to the query based on the identity and length of the sequence covered, returning the serotype and genotype of the reference sequence.

If a complete CDS cannot be obtained through the assembly process, the processed reads are mapped against the same DENV typing database, with Bowtie 2, using the Seq_Typing tool, with similar criteria for coverage and identity to those used with the blast approach. If a type is determined, the consensus sequence obtained follows through to the next step in the workflow. Otherwise, the sample is classified as non-typable and processing is terminated.

#### Phylogeny

All complete DENV CDSs and consensus sequences analysed in a workflow execution are aligned with mafft (version 7.402) [25]. By default, or if the number of samples analysed is less than four, four representative sequences for each DENV serotype (1 to 4) from the National Center for Biotechnology Information (NCBI) are also included in the alignment. The NCBI references included are NC_001477.1 (DENV-1), NC_001474.2 (DENV-2), NC_001475.2 (DENV-3) and NC_002640.1 (DENV-4). The closest reference sequence to each analysed sample in the DENV typing database can also be retrieved and included in the alignment. With the resulting alignment, a maximum-likelihood tree is constructed with RaXML (version 8.2.11) [26].

#### Output and report

The output files of all tools in DEN-IM’s workflow are stored in the ‘results’ folder in the directory of DEN-IM’s execution, as well as the execution log file DEN-IM and for each component.

The HTML report (see Fig. S1), stored in the ‘pipeline_results’ directory, contains all results divided into four sections: report overview, tables, charts and phylogenetic tree. The report overview and all tables allow for the selection, filtering and highlighting of particular samples in the analysis. All tables have information on whether a sample failed or passed the quality control metrics, highlighted by green, yellow or red signs for pass, warning and fail messages, respectively.

The *in silico* typing table contains the serotype and genotype results for each CDS analysed, as well as the identity, coverage and GenBank ID of the closest reference in the DENV typing database. The quality control table shows information regarding the number of raw base pairs and number of reads in the raw input files and the percentage of trimmed reads. The mapping table includes the results for the mapping of the trimmed reads to the DENV mapping database, including the overall alignment rate, and an estimation of the sequence depth including only the DENV reads. For the assembly statistics table, the number of CDSs in each sample, the number of contigs and the number of assembled base pairs generated by either SPAdes or megahit assemblers is included. The number of contigs and assembled base pairs after correction with Pilon is also presented in the table. The assembled contig size distribution scatter plot is available in the chart section, showing the contig size distribution for the Pilon-corrected assembled CDSs.

Lastly, a phylogenetic tree is included, rooted at the midpoint for visualization purposes, and with each tip coloured according to the genotyping results. If the option to retrieve the closest typing reference is selected, these sequences are also included in the tree with respective typing metadata. The tree can be displayed in several conformations provided by the Phylocanvas JavaScript library (http://phylocanvas.net , version 2.8.1) and it is possible to zoom in on or collapse selected branches. The bootstrap support values of the branches can be displayed, and the tree can be exported as a Newick tree file or as a PNG image.

### Software comparison

DEN-IM offers core assembly functionality, leveraging a *de novo* and consensus assembly approach to obtain a full CDS sequence to perform geno- and serotyping, followed by phylogenetic positioning of the samples analysed. This results in a phylogenetic tree showing the genotyping results, presented in an HTML file.

There are several alternative tools, both command line- and online-based, capable of identifying DENV reads and performing assembly (Table 1). VIP and drVM are both stand-alone pipelines, like DEN-IM, and several components overlap with DEN-IM’s, but the retrieval of viral sequences is not targeted for DENV, and no serotyping and genotyping is performed. VIP is, overall, the most similar to DEN-IM by performing viral identification (although it is not specific for DENV), assembly and phylogenetic analysis against the reference database and producing an HTML report with the results obtained. It is not possible to customize VIP′s database to target only DENV sequences with genotyping information. VirusTAP is a web server for the identification of viral reads using the ViPR and IRD databases, or alternatively with the RefSeq Virus database. GenomeDetective is also a web service that provides two tools, one for the assembly of viral sequences from raw data (Virus tool) and another for serotyping and genotyping of DENV fasta sequences (Dengue Typing tool). Both tools need to be run consecutively, with the Virus Tool providing a link to redirect to the Dengue Typing tool when a DENV sequence is identified.

**Table 1.** Comparison of CDEN-IM with different tools for the identification and genotyping of DENV from sequencing data

Tool	Interface	Quality control	DENV read selection	Assembly	DENV sero- and genotyping	Phylogeny	Report
**DEN-IM**	CLI	✓	✓	✓	✓	✓	✓ (all samples)
**VIP**	CLI	✓	✓†	✓	✗	✓	✓
**VirusTAP**	Web	✓	✓†	✓	✗	✗	✓ (one per sample, downloadable)
**drVM**	CLI/GUI*	✓	✓†	✓	✗	✗	✗
**GenomeDetective Virus tool**	Web	✓	✗	✓	✗	✗	✓ (one per sample)
**GenomeDetective Dengue Typing tool**	Web	✗	✗	✗	✓‡	✓§	✓ (one per sample)

*GUI only available on a virtual machine.

†Targeted for viral sequences, but not specific for DENV.

‡Sequence file can be received from GenomeDetective Virus tool, as well as independently uploaded.

§Limited to the positioning of a sample in a tree of static representative isolates.

Of all the tools listed in Table 1, only Genome Detective offers a tool to determine the DENV sero- and genotypes from a fasta sequence, but the need to run their virus identification tool beforehand to obtain a sequence from the raw sequencing data increases the time required to obtain a typing result, especially when a large number of sequences need to be analysed. DEN-IM provides the same information of the Genome Detective Virus Typing tool, with the addition of a phylogenetic tree with all samples analysed plus automatic selection of the closest genomes present in the database (optional) and NCBI DENV references (optional). Moreover, these tools are not open source, so we are unable to compare the methodology used with our own. Additionally, there might be privacy issues in submitting data to external services, such as VirusTAP and GenomeDetective, especially when handling metagenomics data that contain human sequences subjected to strict privacy laws in most countries. Therefore, a stand-alone tool is preferable for these analyses since these can be run in secure local environments. DEN-IM’s main advantage when compared to web-based platforms is the ability to analyse batches of samples in a scalable manner, obtaining a report summarizing all the samples analysed and a phylogeny analysis of all DENV CDSs recovered.

## Results

To evaluate the DEN-IM workflow performance, we analysed three datasets, one containing shotgun metagenomics sequencing data from patient samples (see Table S1), a second with amplicon sequencing data, a set with 106 paired-end samples obtained from Parameswaran *et al.* [27] and another set with 78 single-end samples available under BioProject PRJNA321963, and a third dataset of publicly available sequences, both from amplicon and shotgun metagenomics, containing 45 Chikungunya virus (CHIKV) samples, 66 Zika virus (ZKV) samples and 21 yellow fever virus (YFV) samples (see Table S2). All analyses were executed with the default resources and parameters (available at https://github.com/B-UMMI/DEN-IM). In the shotgun metagenomics and the single-end amplicon sequencing datasets the closest typing reference in the final tree and the NCBI DENV references for each serotype were included in the phylogenetic analysis. The resulting reports for each dataset are available on Figshare at https://doi.org/10.6084/m9.figshare.11316599.v1.

### Shotgun metagenomics dataset

We analysed a dataset containing 22 shotgun metagenomics paired-end short-read Illumina sequencing samples from positive dengue cases, 1 positive control (purified from a DENV culture), 1 negative control (blank) and an *in vitro* spiked sample containing the 4 DENV serotypes (see DEN-IM_Supplemental_material.pdf, Shotgun Metagenomics Sequencing Data). On average, each sample took 7 min to analyse. A total of 75 CPU hours were used to analyse the 25 samples, with a total size of 17 GB. This analysis resulted in 69 GB of data.

The negative control and the 92–1001 sample had no reads after trimming and filtering of low-complexity reads, and so they were removed from further analysis (see Table S3). When mapping to the DENV mapping database, the percentage of DENV reads in the 21 clinical samples, the positive control and the spiked sample passing QC ranged from 0.01 % (sample UCUG0186) to 85.38 % (positive control – PC – sample). After coverage depth estimation, the analysis of the samples 91–0115 and UCUG0186 was terminated due to a low proportion of DENV reads (0.05 and 0.01 %, respectively). They failed to meet the threshold criterion of having an estimated depth of coverage of ≥10× (estimated coverages of 3.17× and 5.65×, respectively). The sequence data from sample 91–0106 only contained 960 DENV reads (0.03 %), but these were successfully assembled into a CDS with an estimated depth of coverage of 14.71×.

In the assembly module, the remaining 19 samples, the spiked sample and the PC were assembled with DEN-IM’s two assembler approach. Twenty-four full CDSs were assembled (see Fig. S2), even in samples originally having DENV read content of as low as 0.03 % of the total reads. Sixteen samples, including the spiked sample and the positive control, were assembled in the first step with the SPAdes assembler, and five were assembled in the second step with the megahit assembler. In the spiked sample, all four CDSs were successfully assembled and recovered.

**Fig. 2.**
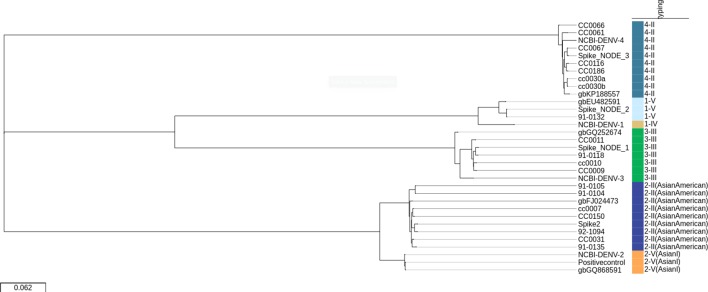
Phylogenetic reconstruction of the shotgun metagenomic dataset. Maximum-likelihood tree in the DEN-IM report for the 24 complete CDSs (*n*=21 samples) obtained with the metagenomics dataset, the respective closest references in the typing database (identified by their GenBank ID) and the NCBI DENV references for each serotype (NCBI-DENV-1, NC_001477.1; NCBI-DENV-2, NC_001474.2; NCBI-DENV-3, NC_001475.2; NCBI-DENV-4, NC_002640.1). The tree is midpoint-rooted for visualization purposes and the scale represents the average substitutions per site. The colours depict the DENV genotyping results.

Serotype and genotype were successfully determined for the 24 DENV CDSs by blast (see Fig. S2). The most common were serotype 2 genotype III (Asian-American) and serotype 4 genotype II, with eight samples each (33 %), followed by serotype 3 genotype III (*n*=5, 21 %), serotype 1 genotype V (*n*=2, 8 %) and serotype 2 genotype V (Asian I) (*n*=1, 4 %). All CDSs recovered and the respective closest reference genome in the typing database were aligned and a maximum-likelihood phylogenetic tree was obtained to visualize the relationship between the samples (Fig. 2). There was a perfect concordance between the serotyping and genotyping results and the major groups in the tree.

Four distinct CDSs were assembled for the spiked sample that resulted in different coverages of each serotype CDS (2032× times coverage for DENV-2, 229× coverage for DENV-1, 76× coverage for DENV-3 and 30× times coverage for DENV-4), in accordance with the ranking order of the real-time RT-PCR results (see DEN-IM_Supplemental_material.pdf, Shotgun Metagenomics Sequencing Data).

### The amplicon sequencing dataset

To validate DEN-IM’s performance in an amplicon sequencing approach, a dataset of 106 paired-end HTS samples of PCR products using primers targeting DENV-3 [27] were analysed (see DEN-IM_Supplemental_material.pdf, Amplicon Sequencing Data). On average, each sample took 5 min to analyse. The 106 samples, 51 GB in size, took 3622 CPU hours to analyse, resulting in 424 GB of data.

No samples failed the quality control block (see Table S4). The proportion of DENV reads ranged from 24.72 % (SRR5821236) to 99.81 % (SRR5821254) of the total processed reads. The samples with less than 70 % DENV DNA were profiled taxonomically with Kraken2 [28] and the minikraken2_v2 database (ftp://ftp.ccb.jhu.edu/pub/data/kraken2_dbs/) and the source of contamination was determined to have come largely from human DNA (see Table S5).

Of the 106 samples, 43 (41 %) managed to assemble a complete CDS sequence (see Table S4), whereas a mapping approach was used for the remaining 63 samples (60 %) and a consensus CDS was generated. For the assembled CDSs, all but one were assembled with megahit after not producing a full CDS with SPAdes. Moreover, pronounced variation in the size of the assembled contigs is evident in the contig size distribution plot (see Fig. S3).

**Fig. 3.**
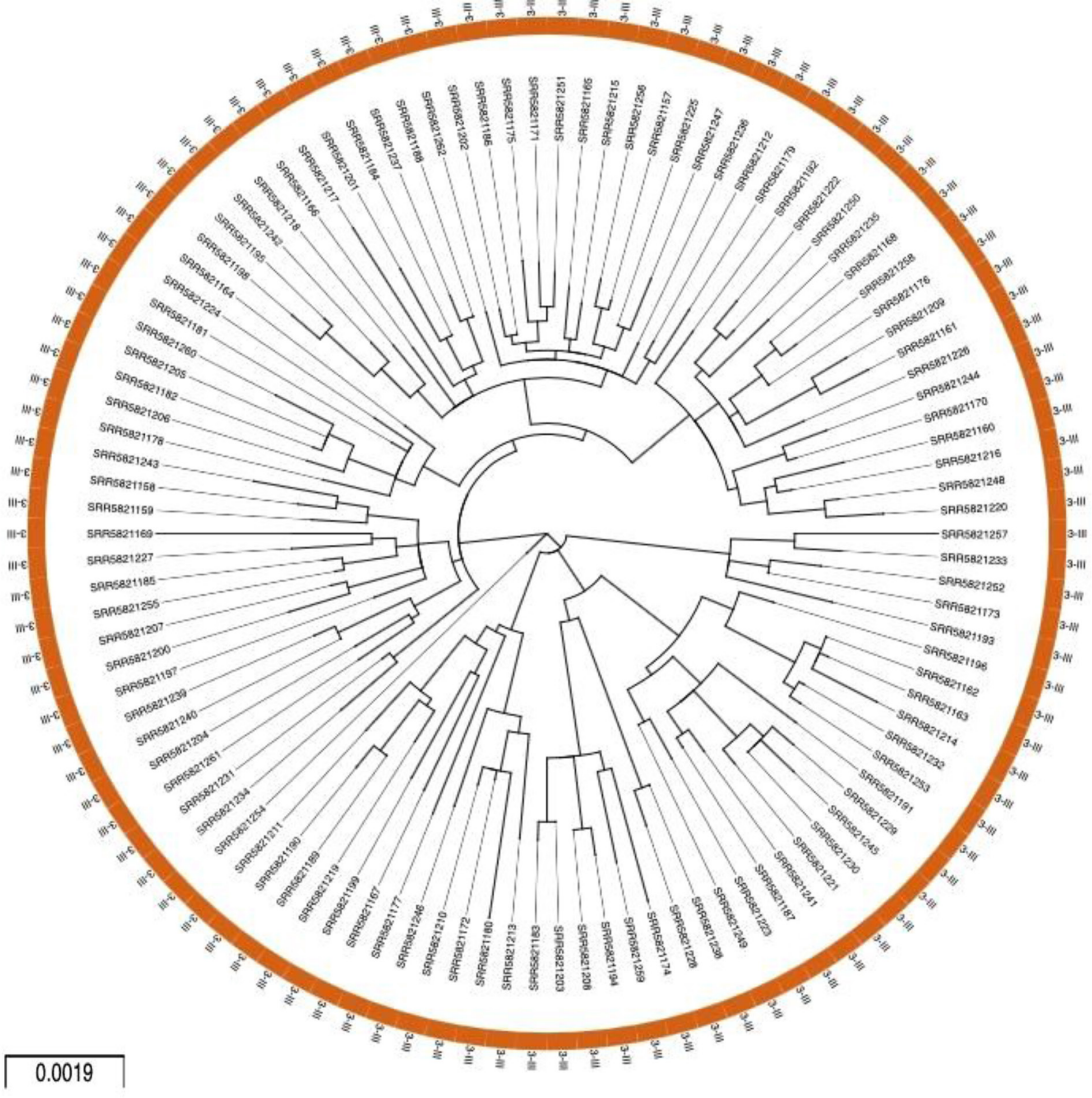
Phylogenetic reconstruction of the paired-end targeted metagenomic dataset. Maximum-likelihood circular tree in the DEN-IM report for the 106 complete CDSs obtained with the targeted metagenomics dataset (*n*=106). All samples belong to serotype 3 genotype III. The scale represents the average substitutions per site.

All 106 CDSs recovered belonged to serotype 3 genotype III. Despite the same classification, the maximum-likelihood tree indicates that there is detectable genetic diversity within the dataset (486 SNPs in 10 237 nucleotides) (Fig. 3).

A second amplicon dataset, containing 78 DENV-1 single-end samples recovered from different *A. aegypti* isofemale hosts were analysed (see DEN-IM_Supplemental_material.pdf, Amplicon Sequencing Data). On average, each sample took 3 min to analyse. The 78 samples, 19 GB in size, took 278 CPU hours to be analysed, resulting in 203 GB of data.

**Fig. 4.**
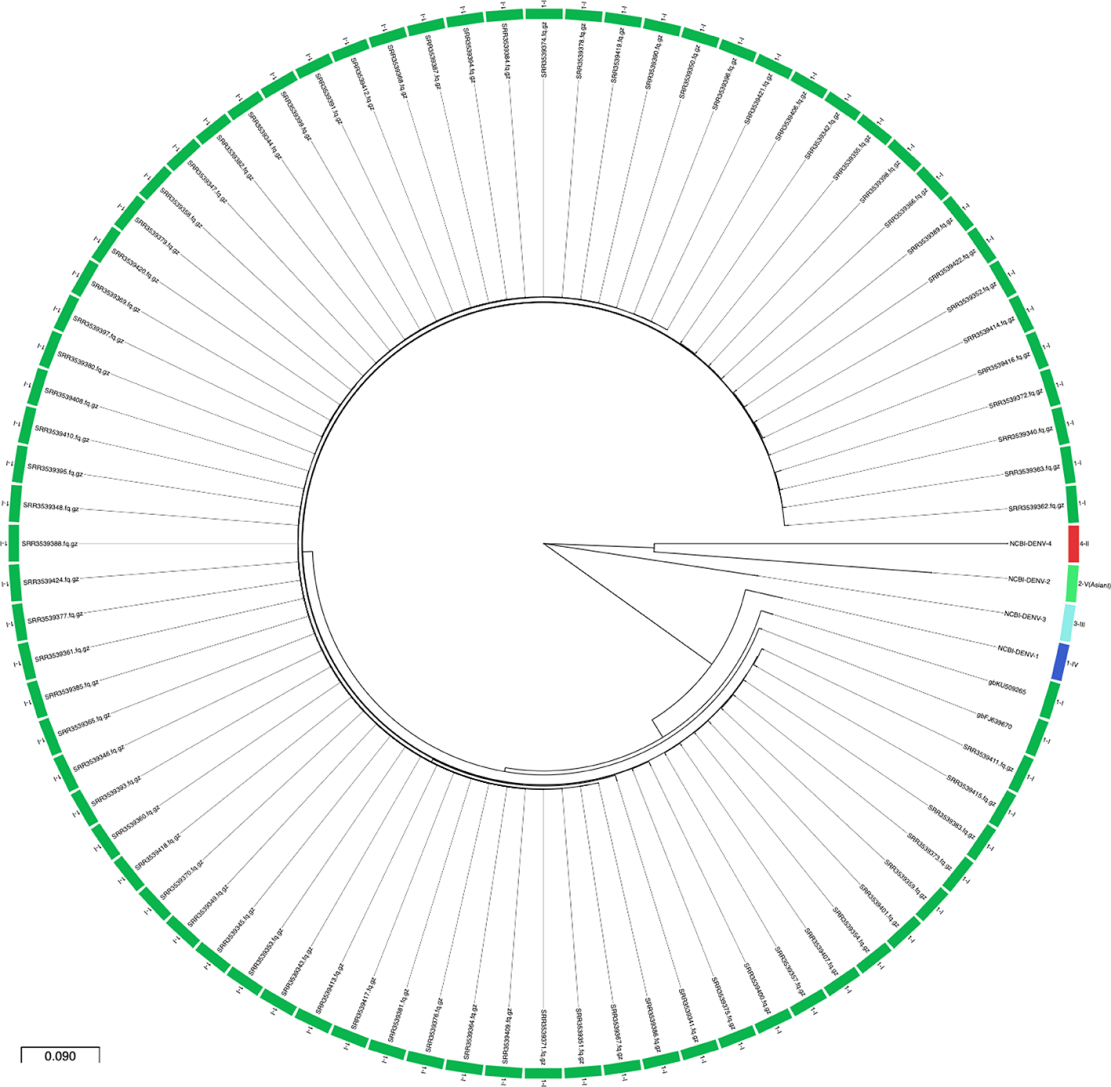
Phylogenetic reconstruction of the single-end targeted metagenomic dataset. Maximum-likelihood circular tree in the DEN-IM report for the 78 complete CDSs obtained with the targeted metagenomics dataset (*n*=78) and the NCBI DENV references for each serotype (NCBI-DENV-1, NC_001477.1; NCBI-DENV-2, NC_001474.2; NCBI-DENV-3, NC_001475.2; NCBI-DENV-4, NC_002640.1). All samples belong to serotype 1 genotype I. The scale represents the average substitutions per site.

No samples failed the quality control block and the proportion of DENV reads ranged from 59 (SRR3539343) to 96 % (SRR3539408) of the total processed reads (see Table S6). Of the 78 samples, 53 (68 %) assembled a complete CDS sequence and in the remaining 25 (32 %) the complete CDS was obtained through mapping. All CDSs recovered, the respective closest reference genome in the typing database and NCBI’s references for each DENV serotype were aligned and a maximum-likelihood phylogenetic tree was obtained (Fig. 4). All 78 samples belonged to serotype 1 genotype I and, similarly to the previous dataset of 106 samples, there was detectable genetic diversity within the dataset (651 SNPs in 10 808 nucleotides, excluding reference sequences).

### The non-DENV arbovirus dataset

In order to evaluate DEN-IM’s specificity to DENV sequences, a third dataset of publicly available sequences of arbovirus other than DENV, from both amplicon and shotgun metagenomics, was analysed containing 45 CHIKV samples, 66 ZKV samples and 21 YFV samples (see Table S2). All 132 samples failed DEN-IM’s workflow, 16 due to insufficient sequencing data remaining after quality trimming, and the remaining 116 due to very low estimated coverage of the DENV genome (less than 0.01×), as expected.

### Conclusion

We have successfully analysed two DENV datasets, one comprising 25 shotgun metagenomics sequencing samples and another comprising 106 paired-end and 78 single-end targeted metagenomics samples.

In the first dataset, we recovered 24 CDSs from 19 clinical samples, including a spiked sample and a positive control that were correctly serotyped and genotyped. Besides the negative control, three samples did not return typing information due to failing quality checks.

The proportion of DENV reads in the metagenomics samples was highly variable. This may reflect the viral load in patients in which DENV was detected by real-time RT-PCR. In the spiked sample, containing four distinct DENV serotypes, all four were correctly detected despite not being present in equal concentrations, highlighting the potential of the DEN-IM workflow to accurately detect and recover multiple DENV genomes from samples with DENV co-infection, even if the serotypes are present in low abundance. Indeed, recent studies from areas of high endemicity suggest that co-infection with multiple DENV serotypes may occur frequently [29, 30] and the co-circulation of different DENV strains of the same serotype, but distinct genotypes, in these areas [29] raises the possibility of simultaneous infection with more than one genotype.

When analysing the 106 paired-end targeted metagenomics dataset, only 43 CDS samples were *de novo* assembled. For the remaining 63 samples, consensus sequences were obtained through mapping. In all samples DENV 3-III was correctly identified. Similar results were obtained for the 78 single-end samples where 53 CDS were *de novo* assembled, and 25 consensus sequences were obtained through mapping. All samples were identified as DENV-1 I. These two datasets demonstrate the success of DEN-IM’s two-pronged approach of combining assembler and mapping. DEN-IM’s specificity was shown when it found no false-positive results when analysing a dataset containing arboviruses other than DENV.

DEN-IM is built with modularity and containerization as keystones, leveraging the parallelization of processes and guaranteeing reproducible analyses across platforms. The modular design allows for new modules to be easily added and tools that become outdated to be easily updated, ensuring DEN-IM’s sustainability. The software versions are also described in the Nextflow script and configuration files, and in the Docker files for each container, allowing the traceability of each step of data processing.

Having been developed in Nextflow, DEN-IM runs on any UNIX-like system and provides out-of-the-box support for several job schedulers (e.g. PBS, SGE, SLURM) and integration with containerized software such as Docker or Singularity. While it has been developed to be ready to use by non-experts, not requiring any software installation or parameter tuning, it can still be easily customized through the configuration files.

The interactive HTML reports (see Fig. S1) provide an intuitive platform for data exploration, allowing the user to highlight specific samples, filter and reorder the data tables, and export the plots as needed.

Together with the workflow and software containers, a database containing 3858 complete DENV genomes for DENV sequence retrieval and a subset database with 161 curated DENV genomes for serotyping and genotyping are provided. While constructing these databases, the obstacles reported by Cuypers *et al.* [31] were apparent, namely the lack of formal definition of a DENV genotype and the lack of a standardized classification procedure that could assign sequences to a previously defined genotypic/sub-genotypic clade [31]. Discrepancies between the phylogenetic relationship and the genotype assignment were frequent and, throughout this study, the classification of some strains within the ViPR database [32] was updated. As suggested previously [31], further evaluation of DENV classification will benefit future research and investigation into the population dynamics of this virus. Our typing approach was designed to use the currently accepted DENV classification. However, DEN-IM can be easily modified if a new DENV classification system is to be established in the future.

DEN-IM provides a user-friendly workflow that makes it possible to analyse short-read raw sequencing data from shotgun or targeted metagenomics for the presence, typing and phylogenetic analysis of DENV. The use of containerized workflows, together with shareable reports, will allow an easier comparison of results globally, promoting collaborations that can benefit the populations where DENV is endemic. The DEN-IM source code is freely available in the DEN-IM GitHub repository (https://github.com/B-UMMI/DEN-IM), which includes a wiki with full documentation and easy-to-follow instructions.

## Data bibliography

1. Catarina Inês Mendes. DEN-IM supplemental material and tables are deposited at Figshare with DOI; https://doi.org/10.6084/m9.figshare.11316599.v1.

2. Catarina Inês Mendes. DEN-IM reports for the analysed datasets tables are deposited at Figshare with DOI; https://doi.org/10.6084/m9.figshare.11316599.v1.

3. Catarina Inês Mendes. Phylogeny inference trees for the dengue virus typing database are deposited at Figshare with DOI; https://doi.org/10.6084/m9.figshare.11316599.v1.

4. Catarina Inês Mendes. Code for the DEN-IM workflow (https://github.com/B-UMMI/DEN-IM).
